# On the Method Employed for Disgorging Leeches to Render Them Fit for Use after Previous Application

**Published:** 1848-06

**Authors:** 


*On the Methods Employed for Disgorging Leeches to render them fit for use after previous Application.* By MM. Soubeiran and Bouchardat.—A cavity was made in a firm soil, and filled with white unctuous clay, of such a consistence that the leeches might easily penetrate it, taking the precaution, however, not to have it too soft. The soil had a slight inclination to enable the water to run off through a grating placed at the lowest part. In this manner the clay was moistened and not covered with water, except at the lower part. The clay being thus arranged, was inundated with water every day so as to wash it and moisten it slightly. Every day, also, the dead leeches, that rose to the surface were removed. To prevent the escape of the leeches, the brink was surrounded with bricks, cemented with Roman cement, about a foot in height; it was then covered with a wooden frame that over-reached it about one foot, and to which were fixed, by means of nails, some pieces of cloth about two and a half or three inches in breadth, unravelled at the lower part to the height of three-quarters of an inch or an inch, and this was found to be a sufficient obstacle to the escape of the leeches. The aperture in the soil was twenty inches square, and about a foot deep, 6,500 leeches were placed in it; the experiment was commenced in the month of December; in the month of June following, the leeches were taken out, after allowing the clay to dry to a firm paste. They were found in groups of two to six together, in small crevices, surrounded with a greenish matter. They were very lively, and were placed in water, which they immediately tinged green. In two or three days they were equal in appearance to the best new leeches, and superior to them in quality, for they all took quickly, and remained a longer time on the patient. From this it would appear that the question was decided; but we found afterwards, in operating on larger numbers, sufficient for the consumption of the Hôtel Dieu, that we were less fortunate. Two causes tended to this unfavourable result; on the one hand, gorged leeches are more liable to disease than others; on the other, the blood that they discharge becomes by its decomposition an incessant cause of their destruction. Profiting by these failures, we then tried only a limited number of leeches, but notwithstanding the greatest care, we could only employ a fifth, or at most a fourth, of the number originally taken. Still, however, we had no doubt as to the possibility of ultimate success, and in this we were confirmed by the information obtained from M. Lesson, of the Hospital at Rochefort. The arrangement there consists of two reservoirs or vessels of cut stone.

The bottom of these vessels is coated with a bed of blue clay about a foot deep. In the centre is a beechwood frame, filled with the same clay in which are placed some aquatic plants; this mound is intended to serve as a protection for the leeches, which penetrate deeply into it, and produce their cocoons. The plants contribute to keep the water, which runs in at the top, fresh and supplied with air, whilst the thick water rendered impure with the blood, escapes by the pipe at the lower part of the vessel. Each vessel will contain 20,000 leeches, and in two years they have paid for the cost of their construction. Before placing the leeches in these vessels, M. Lesson immerses them in a small quantity of water in vessels running obliquely to the bottom. This water, replaced frequently by fresh, in the course of eight or ten days cleanses them from the external blood, and is charged with a quantity of mucous matter. The leech assimilates by degrees, or rejects the blood it has sucked, and after this time they are put in the reservoir, where they lose gradually their torpor under the influence of fresh water, comparative abstinence, and the elements of their natural habitations. At the hospital at Metz they have had for more than eight years a pond, in which they catch on an average from

16,000 to 18,000 leeches annually. This pond has the greatest resemblance to marshes and lakes. The water runs off but slowly, and is supplied anew by a stream running from one of the leats of the town. This reservoir is not cemented or coated with clay; the bottom is covered with slime aud mud, in which the leeches obtain their support. Several aquatic plants are also disseminated in it. In spite of all the precautions taken, however, not more than a third of the gorged leeches that had been deposited there were retaken.

After numerous experiments, we give the preference to the method of pressure, if carefully managed, and we have obtained at the Hôtel Dieu, most satisfactory results, which have also been arrived at by others, engaged in the investigation of the subject. We admit, in fact, that of all the methods we have tried, the one presenting the greatest advantages is that of slight pressure between the fingers. This process, which we at first rejected, and which in its most simple form we still consider as impracticable, is entirely changed in character when submitted to two important modifications: First, the tendency of leeches to disgorge under a slight excitement; secondly, the immersion of these animals in warm water, which keeps up a gentle and necessary stimulus, and gives to the blood a fluidity that renders its discharge more easy. These two conditions are indispensable in practice. As has been well remarked by M. Huzard, in his report, as well as by those who have considered the subject, the leeches, when pressed, contract the muscles of the mouth strongly, and numbers of them refuse so obstinately to return the blood, that their internal structure is destroyed before they will discharge it. The disgorged leeches are allowed to remain at rest in fresh water for some days, they are then applied again, and subjected afterwards to a second disgorgement. Many, however, are so debilitated that they bite without drawing much blood. Custom enables us to judge with much certainty, from their external appearance, whether they are capable of being immediately again employed, and if they are not, they are placed in small artificial ponds, where they may continue at rest some time. Some will undergo two, three, or even more disgorgements, without being placed in the ponds.

*Disgorgement of the Leeches.*—This should be done on the same day in which they are applied. A dozen are thrown into a solution of 16 parts salt and 100 parts water. Each leech should be taken by its posterior extremity, and dipped in the water, which should be warm, but not more so than the hand can bear; it should then be passed lightly between the fingers, when it will return without difficulty the blood it has taken. The leeches should then be set by in vessels containing fresh water, which should be renewed every twenty-four hours. In the course of eight or ten days, they will be fit for further use, and answer equally as well as new ones. When again used they are to be treated in the same manner; and if it is found that they will not bear a third application, they are placed in the ponds.

*Description of the Artificial Ponds.*—They consist of stone vessels, internally coated over with Roman cement. These are filled with water, which is renewed immediately that it exhibits an alkaline reaction. This is an important condition, for alkaline matter is perhaps the most fatal to leeches. One of these vessels will be sufficient for the annual use of 50,000 leeches. Each vessel is divided into three compartments of the following dimensions: length, 39 feet $4\frac{1}{2}$ inches; breadth, 5 feet 7 inches; depth 2 feet 2 inches. The bottom of the vessel is covered with a stratum of softened clay $15\frac{1}{2}$ inches thick, in which some aquatic plants and grasses are planted. The leeches bury themselves in the moist clay, from which they come out again when recovered. A constant stream of water is allowed to flow slowly through the vessel. There is very little mortality in these ponds, because its principal cause, the putrefaction of the blood discharged, is removed.

The construction of these vessels differs; sometimes they are lined with zinc, but the presence of vegetables, is, we think, always advantageous.—*Ibid, from Repertoire de Pharmacie.*

